# Direct Real-Time Measurement of Intra-Oocyte Nitric Oxide Concentration *In Vivo*

**DOI:** 10.1371/journal.pone.0098720

**Published:** 2014-06-02

**Authors:** Pravin T. Goud, Anuradha P. Goud, Tohid Najafi, Bernard Gonik, Michael P. Diamond, Ghassan M. Saed, Xueji Zhang, Husam M. Abu-Soud

**Affiliations:** 1 Division of Reproductive Endocrinology and Infertility, Department of Obstetrics & Gynecology, The C. S. Mott Center for Human Growth and Development, Wayne State University, Detroit, Michigan, United States of America; 2 Division of Reproductive Endocrinology and Infertility, Department of Obstetrics and Gynecology, University of California Davis, Sacramento, California, United States of America; 3 California IVF Fertility Center, Davis and Sacramento, California, United States of America; 4 California National Primate Research Center, University California Davis, Davis, California, United States of America; 5 Department of Obstetrics & Gynecology, Georgia Regents University, Augusta, Georgia, United States of America; 6 World Precision Instruments, Sarasota, Florida, United States of America; 7 Research Center for Bioengineering & Sensing Technology, University of Science & Technology, Beijing, P.R. China; 8 Department of Biochemistry and Molecular Biology, Wayne State University, School of Medicine, Detroit, Michigan, United States of America; Institute of Zoology, Chinese Academy of Sciences, China

## Abstract

Nitric oxide (NO) is reported to play significant a role in oocyte activation and maturation, implantation, and early embryonic development. Previously we have shown that NO forms an important component of the oocyte microenvironment, and functions effectively to delay oocyte aging. Thus, precise information about intra-oocyte NO concentrations [NO] will result in designing more accurate treatment plans in assisted reproduction. In this work, the direct, real-time and quantitative intra-oocyte [NO] was measured utilizing an L-shaped amperometric integrated NO-selective electrode. This method not only provides an elegant and convenient approach to real-time the measurement of NO in physiological environments, but also mimics the loss of NO caused by rapid NO diffusion combined with its reactivity in the biological milieu. This experiment suggests that the NO levels of oocytes obtained from young animals are significantly higher than those of oocytes obtained from old animals. Additionally the NO levels stay constant during the measurements; however, the intra-oocyte [NO] is reduced significantly (70–75% reduction) in response to L-NAME incubation, suggesting that NO measurements are truly NOS based rather than caused by an unknown interfering substance in our system. We believe this first demonstration of the direct quantitative measurement of [NO] in situ in an intact cellular complex should be useful in tracking real-time and rapid changes at nanomolar levels. Moreover, this finding confirms and extends our previous work showing that supplementation with NO delays the oocyte aging process.

## Introduction

Nitric oxide (NO) is a ubiquitous signaling molecule that comprises an important component of the oocyte microenvironment, and plays a significant role in the developing oocyte from oogenesis to fertilization [1]. Nitric oxide is typically generated from L-arginine (L-Arg), oxygen (O_2_), tetrahydrobiopterin (H_4_B), and NADPH by the three distinct isoforms of NO synthase (NOS): neuronal (nNOS), inducible (iNOS), and endothelial (eNOS). In the deficiency of L-Arg and/or H_4_B, reduction of NOS by NADPH results in toxicity by generation of superoxide (O_2_•-), a highly reactive free radical, instead of NO [2]. The biological effects of NO are governed in part, by its intrinsic instability, reactivity, lipophilicity, and affinity toward iron; these characteristic features make it ideal for both signal transduction and defense [3]. We have previously shown the critical role of this molecule in the female reproductive system, in that exogenously added NO or supplementation with NOS natural substrate L-Arg and the cofactor H_4_B, delays oocyte aging whereas treatment with L-NG-nitroarginine methyl ester (L-NAME), a NOS inhibitor, deteriorates oocyte quality and accelerates the aging process [4], [5]. These findings suggest that direct and precise intracellular measurements of NO to monitor rapid alterations in NOS activity are of high demand. However, for samples such as oocytes that face limitations in volume and/or number, the sensitivity of a technique to directly measure NO concentration [NO] is critically important [6].

Different NOS isoforms play different roles in various reproductive events. The participation of NO in mediating sGC signaling pathway in oocytes suggests the involvement of the three NOS isoforms during normal fertilization and development. Previously, we have shown that inhibition of NO synthesis or downstream mediators significantly worsened fertilization and development to the blastocyst stage, and enhanced arrest and apoptosis in the embryos. Optimal concentrations of intra-oocyte NO are required for preserving the fertilization quality. Thus, information about the bioavailability of NO during this process is significantly important and requires exact monitoring of the changes in NO levels during the post ovulatory period. Direct intra-oocyte [NO] measurements to track short-term changes are needed to characterize and quantify the effects of NO alteration on oocyte quality.

Several direct and indirect methods for the measurement of [NO] and its redox related species [i.e., nitrite (NO_2_^-^) and nitrate (NO_3_^-^)] both *in vivo* and *in vitro* with exclusive characteristics and different ways of function have been developed over the past two decades [7]. The most frequently used techniques include: absorbance, chemiluminescence and fluorescence assays, capillary electrophoresis, electron paramagnetic resonance spectroscopy (EPR), electrochemistry, and high-performance liquid chromatography. Despite the fact that each technique has a particular individual strength, all of the techniques display weaknesses ranging from a lack of sensitivity or specificity, to interference from factors that commonly exist in biological systems. Thus, the choice of a technique is dependent upon the desired applications. For example, the Griess reaction is the most widely used and measures endogenous NO_2_^-^/NO_3_^-^, the oxidation end products of NO [8]. The low sensitivity of this method especially in the real-time detection of constitutive NO resulted in development of a chemiluminescence technique, which is approximately 100-fold more sensitive [9]. The chemiluminescence assay measures NO in the gaseous state based on the detection of light emitted upon NO's reaction with ozone. This method is highly sensitive and ideal to detect and quantitate gaseous NO, specifically in pulmonary system, but its exclusive response to gases has made it non-compatible for many other systems in which NO is not in the gaseous form. Furthermore, some of the detected nitric anions may originate from spontaneous compound decomposition or from alternative metabolic pathways in addition to NOS activity [6]. However the luminol/H_2_O_2_ chemiluminescence technique has been utilized to detect [NO] in solutions [10]. The oxyhemoglobin assay is a viable, specific and simple alternative with high sensitivity in nanomolar range [11]. This method estimates [NO] based on the amount of NO_3_^-^ (and methemoglobin) generated from the reaction of NO with oxyhemoglobin at near diffusion rate. The oxyhemoglobin assay, with its great sensitivity, specificity, and economic advantage has been modified to be adapted for high sample throughput and small sample volumes. However, there are several factors such as temperature, pH, and other redox-active or heme-containing proteins which can interfere with the results [12]. EPR uses the spectrum generated from conjugation of both endogenous and exogenous NO with a specific metal complex, a so called “spin trap” to quantitively analyze the [NO] [13]. Moreover, specific organic and metal based fluorescent indicators are able to detect NO with high sensitivity through fluorescent imaging techniques. Cupric (Cu^2+^) fluorophores utilize modified versions of these procedures which work based on fluorescent emission by NO^+^, resulting from the reduction of Cu^2+^ by NO [14].

Accordingly, the use of an electrochemical NO sensor provides an elegant and convenient approach to measure NO in real-time in biological samples. Currently, these sensors provide the only highly sensitive means, which provides the ability to measure [NO] continuously and directly within the tissues [15]. In the following protocol, we determined intra-oocyte [NO] in vivo, by modification of L-shaped amperometrically integrated NO-selective electrodes. These electrodes are not affected by ascorbate, NO_2_^-^/NO_3_^-^, L-Arg, or dopamine [16]. Although reactive lengths were as short as 5 µm, the electrode still responds rapidly.

## Materials and Methods

### Study Design

The study involved the use of oocytes obtained from super-ovulated 8–14 week-old (young) and 40–45 week old retired breeder (old) B6D2F1 mice (n = 19 for each group), which was approved by Wayne State University's Animal Investigation Committee.

### Equipment

**Figure 1:**
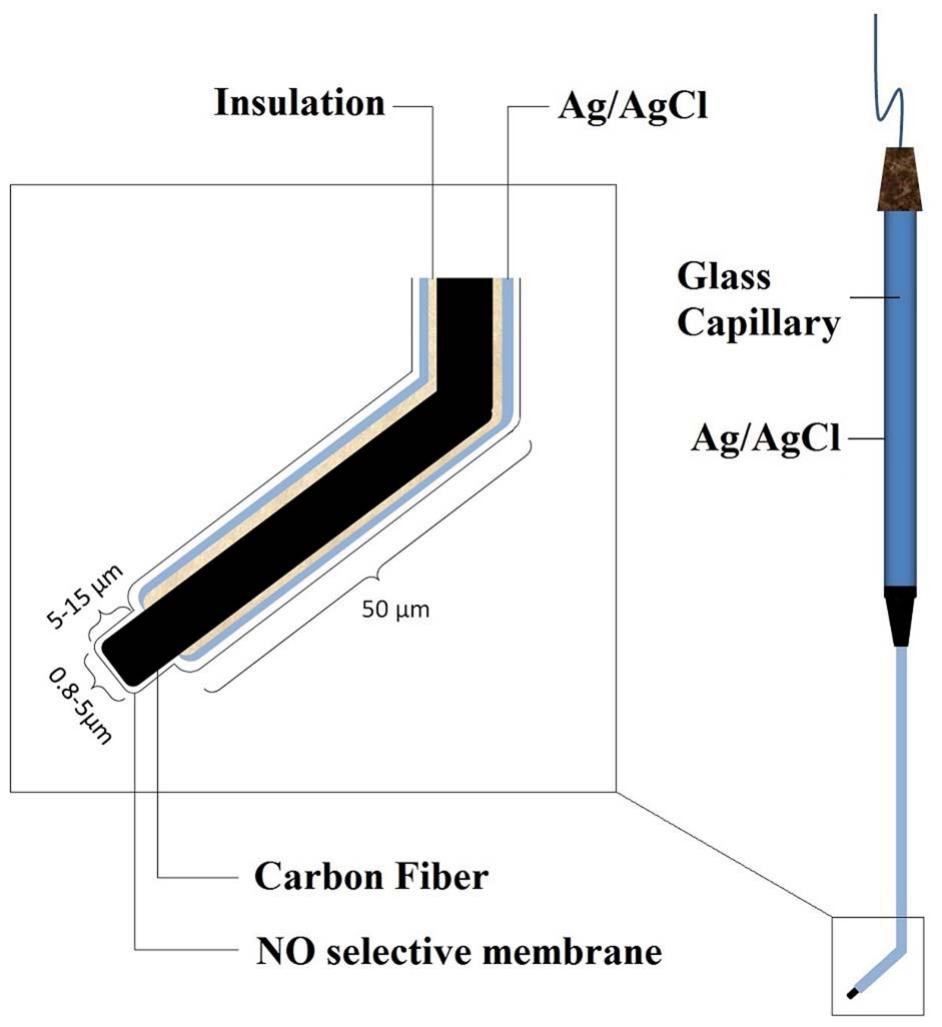
Basic image of the NO-selective electrode structure used in this study. The electrode was provided by the World Precision Instrument.

NO measurements were performed utilizing an Apollo-4000 NO meter (World Precision Instruments, Sarasota, FL) equipped with an L-shaped NO electrode (ISO-NOP). The basic features of this electrode are shown in Figure 1. The absolute NO-reactive part of the needle electrode compromises its proximal 5–15 µm with a diameter of 0.8–5 µm, which is insulated by glass and has 0.5 nM limit of detection of nitric oxide. Ag-AgCl acts as a reference electrode and Nafion, a cation exchanger, which is also used to make NO-selective membranes, functions as a barrier to distinguish NO_2_^-^ derived from NO oxidation from that produced electrochemically.

### Instrument Design

The electrode used in this study had a 10 µm NO reactive part and a 2 µm diameter. The instrument calibration was performed at 37°C. The electrode was equilibrated and polarized along the recommendations of the manufacturing company (WPI). Special microtools were utilized for this procedure; these pipettes were made to meet the requirements of the measurements. For calibration, S-nitroso-N-acetyl-d,l-penicillamine (SNAP) was used in combination with the catalyst Cuprous (I) chloride (CuCl) to generate a known amount of NO in solution [17]. The calibration curve was constructed by plotting the signal output versus the concentration of the SNAP added at that time. A manipulated exclusive form of the aforementioned electrode was designed as follows. We made a 45^o^ angle curvature in the insulated part of electrode before insertion. The major aim of this approach is to increase solidification of the electrodes; because this extremely fragile electrode could be easily broken if it is directly inserted in oocyte longitudinally. This curvature facilitates easy placement into the oocyte and also acts as a barrier preventing excessive movement of the electrode into the ooplasm and inefficient placement of the electrode or puncture of the opposite side of oolemma. Also, handling and support of the oocyte during electrode insertion becomes considerably convenient. Since the diameter of the oocyte during capacitation and maturation varies between 30–80 µm, the curvature was made at a distance of 50 µm proximal to the tip. By this method the response of the electrode is relatively fast due to its close proximity to the source of NO.

### NO-selective electrode calibration

Cuprous (I) chloride, CuCl, was used as the active catalyst for the 100% conversion of SNAP to NO [18]. To eliminate the oxidation of Cu (I) to Cu (II), all the calibration processes were carried out in the dark and in anaerobic atmosphere in pure water or HTF media (Irvine scientific, CA), pH 7.0, at 37+0.2 °C. The anaerobic CuCl solutions were prepared using an all-glass vacuum system [19]. CuCl [150 mg] was placed in a septum-sealed two neck round bottom flask that was equipped with a quick-fit joint for attachment to a vacuum system, and made anaerobic by several cycles of evacuation and equilibration with nitrogen gas. Purification of the nitrogen gas was performed by passing the gas over a heated column of BASF catalyst R3-11 (Chemical Dynamics CorpJKontes Glass Co.). Pure water (HPLC grade, Sigma) or HTF media was deoxygenated and gassed with nitrogen in a separate vessel. Five hundred ml of the pure water/media was transferred to the anaerobic CuCl using a gas-tight syringe and the solution was maintained in the dark under positive N_2_ pressure. Using the same methods, a deoxygenated SNAP solution was also prepared in the presence of 5 mg EDTA in 250 ml pure water (HPLC grade, Sigma) and kept in the dark at 4 °C before usage. Proper dilutions had been made to allow using 3–5 µl to achieve the desired final concentrations.

10 ml of the Cu (I) solution was transferred using a gas-tight syringe to an anaerobic vial with a small stirring bar. The NO-electrode was immersed into the solution, the vial placed over a plate stirrer, and the electrode allowed to stabilize for 3–5 min. Aliquots of different concentrations of SNAP were added to the Cu (I) solution mixture. The current (pA) output from the ISO-NO was increased rapidly. Within a few seconds the response reached a plateau and the second aliquot of SNAP was then added. Successive additions of the remaining aliquots of SNAP were made in a similar way. The concentration of SNAP (and hence NO produced) in the stock solution was calculated as follows: 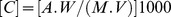



Where C is the concentration of SNAP in µM, A is the purity of SNAP, W is weight of SNAP in mg, M is the formula weight of SNAP, and V is volume of the solution in liters.

**Figure 2:**
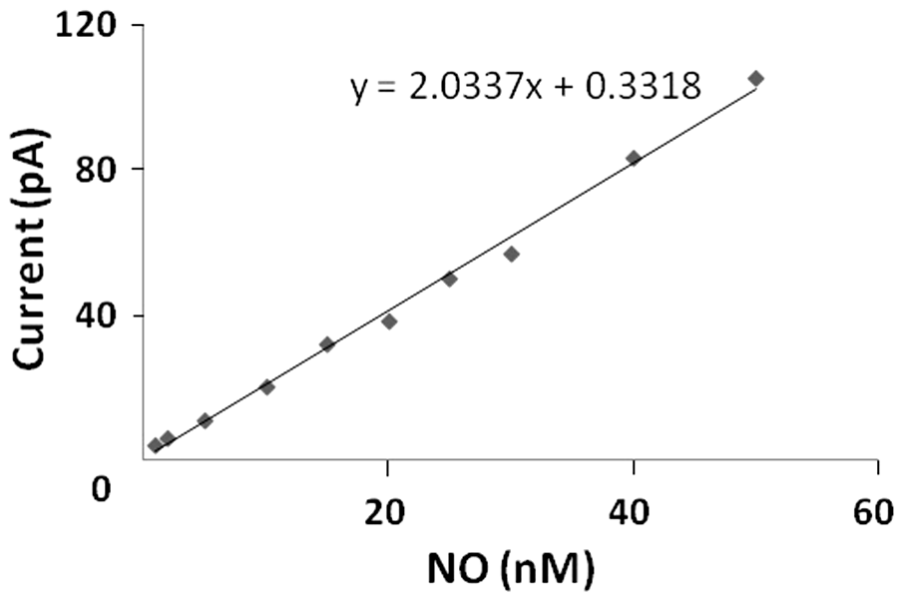
The resulting calibration curve of the responses of an NO selective electrode to the additions of SNAP into CuCl under an anaerobic atmosphere, at 37°C.

As shown in Figure 2 a calibration curve was constructed by plotting the signal output (pA) vs concentration (nM) of SNAP/NO. The calibration curve was also confirmed by the addition of known amount of NO from NO-saturated stock solution to the media [20]. The slope of the calibration curves carried out in water and HTF media were identical. The slope was then determined and entered into the Apollo 4000 software program to observe data in nM concentration mode.

### Procedure for in situ Measurement

With the use of the oocyte media (PBS buffer) surface as the "zero point," the NO electrode tip was inserted directly into the ampulla of the oviduct after making a tiny puncture with a sharpened pulled glass microcapillary. Effective insertion of electrode into the oocyte was monitored under microscopic surveillance. For the NO measurement, the picoampere difference was recorded, and the stabl
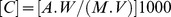
e intra-oocyte reading was taken as the NO signal. The corresponding nanomolar concentration was read off the mean of the bracketed SNAP- CuCl calibration curve. In separate experiments, oocytes were processed to remove cumulus cells by 0.1% (w/v) hyaluronidase treatment followed by gentle pipetting and a micromanipulation procedure in which the zona pellucida (ZP) was slit open using a partial zona dissection (PZD) micropipette, and a 5 µm diameter probe was inserted through the ZP opening deep into the ooplasm. The oolemma was broken after deep invigilation using a technique similar to Intra-cytoplasmic sperm injection (ICSI). Measurements were then performed. The picoampere difference for the NO signal and the corresponding nanomolar difference in concentration were read off the mean of the NO calibration curves. The micromanipulation procedure was performed using a three-axis hanging joystick oil hydraulic manipulator (Narishige, Tokyo, Japan).

### Statistical Analysis

Independent t-tests were performed in the following comparisons by use of SPSS software version 22: control vs. treatment for pooled age groups, young and old for control groups, young vs. old for pooled treatment groups and control vs. treatment for either age group.

## Results

**Figure 3:**
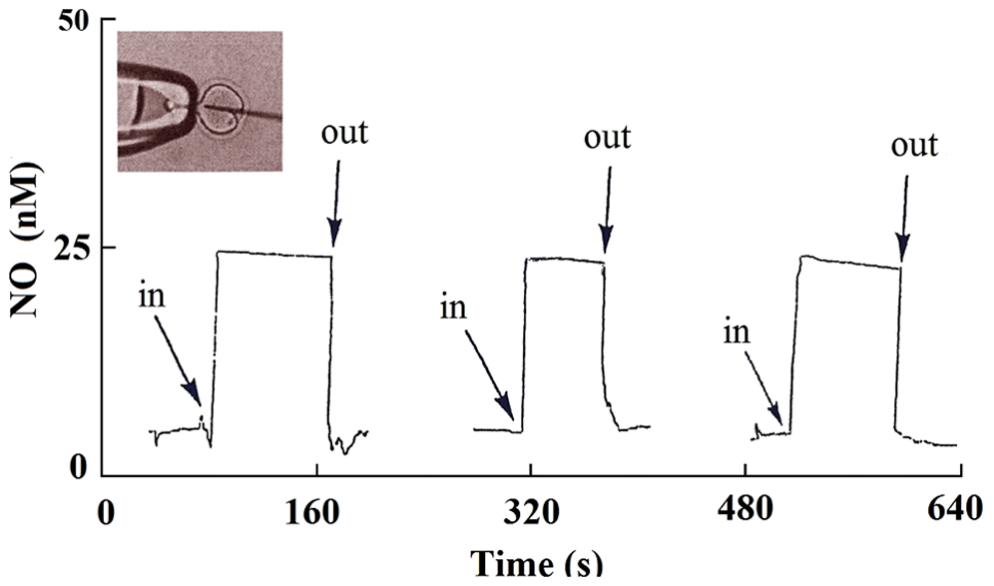
Real-time measurements of intra-oocyte NO concentration utilizing NO-selective electrode. With the use of the oocyte media (PBS buffer) surface as the “zero point”, the NO electrode tip was inserted directly into the ooplasm. The picoampere differences were recorded and the stable intra-oocyte reading was taken as the NO signal. The arrows show the time of insertion and withdrawal from the 3 different oocytes. The inset is when the oocyte ZP was slit open using a PZD micropipette, and a 5 µm diameter probe was inserted deep into the ooplasm, The oolemma was broken after deep invagination using conventional ICSI technique.

**Figure 4:**
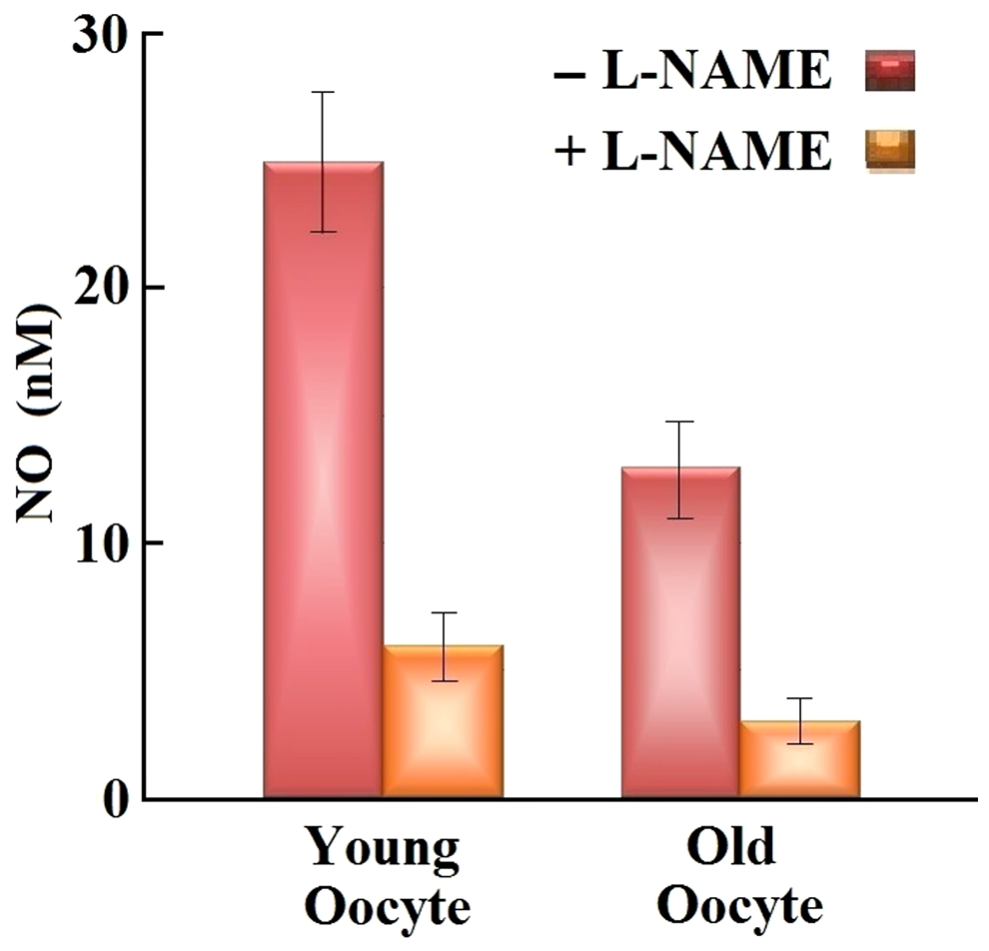
Effect of L-NAME, an NOS inhibitor, on intra-oocyte NO concentration (n = 25 for each group). The error bars represents the standard errors of mean.

In this study, we inserted an NO sensitive electrode tip directly into the ampulla of oviduct and recorded the picoampere differences and nanomolar concentrations of NO. Figure 3 shows the results for real-time profiling of intra-oocyte [NO] *in vivo*. The arrows in this Figure show the time of insertion and withdrawal from the several individual oocytes. The insertion of the NO electrode into the oocyte during NO measurements is shown in the inset of Figure 3. For oocytes that were incubated with 50 µM L-NAME, for 20–30 min, the [NO] fell significantly by 70–75% compared with non-treated oocytes (Figure 4). These results confirm that the NO measured is specifically due to NOS activity rather other different possible sources. All mentioned comparisons made using t-test for equality of means and Levene's test for equality of variances showed significant differences (P = 0.015 for control for both age groups, and P = 0.000 for all other groups). Standard errors of the means were calculated for statistical demonstration.

The viability of oocytes was assessed visually under 600× magnification using Nomarsky contrast before, during and after the probe insertion procedure. Parameters assessed included intactness of shape, membrane turgidity and cytoplasmic characteristics immediately following the probe insertion as well as after culture at 37°C under 5% CO_2_ in air for 1 hour. Oocytes undergoing lysis generally have dark cytoplasm, altered shape and loss of membrane turgidity as noted in mouse oocytes that undergo lysis after any micromanipulation [5], [21], [22]. We did not see any of these signs among the oocytes. Furthermore, readings of NO levels were steady during the entire time of probe insertion.

## Discussion

Our current results introduce a very important tool for direct quantitative measurement of the intracellular levels of NO, a molecule that affects biological and physiological function of the oocyte, utilizing L-shaped 5–15 µm tip NO-selective electrodes. Our findings suggest that the [NO] of oocytes obtained from young animals (25+3 nM; n = 25) are significantly higher than those of oocytes obtained from old animals (13+2 nM; n = 25). While the NO levels stay constant during measurements, the intra-oocyte [NO] was reduced significantly (70–75% reduction) in response to L-NAME incubation, suggesting that NO measurements were truly NOS based rather than caused by an unknown interfering substance in our system. These findings confirm and extend our previous work, which showed that supplementation with NO delays the oocyte aging process by diminution of oocyte ooplasmic microtubule dynamics (OMD), cortical granule (CG) loss, ZP dissolution timing and improves spindle and chromosomal integrity [4]. We believe this is the first demonstration of the direct quantitative measurement of [NO] in situ in an intact cellular complex, which will be useful in validating previous methods, as well as tracking real-time and rapid changes at nanomolar levels. These findings, in part, contribute to our establishment of the hypothesis that NO plays an essential role in maintaining oocyte quality.

The decrease in NO levels in oocytes obtained from old animals could be explained by age-related enhancement in oxidative stress and the decrease in antioxidant machinery. Alternatively, enhancement of arginase II (an enzyme that convert L-Arg to Orn) activity and/or NOS uncoupling (lower levels of H_4_B and/or L-Arg levels) could lower NO levels [2]. Under these circumstances, the NOS heme iron-catalyzed oxygen reduction still occurs but results in O_2_^•-^ formation instead of NO synthesis. Our current results are consistent with our already published results that showed that L-Arg, NO, and/or H_4_B supplementation to oocytes obtained from young and old animals improves oocyte quality and prevents oocyte aging [4].

Thomas et al. have shown that rapid NO diffusion (3300 µm^2^ s^−1^) combined with its reactivity in the biological milieu, contributes to [NO] diminishing while the distance from the point of generation is increasing [23]. Utilizing a porphyrinic sensor, Brovkovych et al., have shown an exponential decrease in the [NO] with increasing distance from the source by stimulation of NO release from a single endothelial cell [24]. Accordingly, their experiments detected a concentration of approximately 950 nM of NO at the cell surface while NO became undetectable at distances >50 µm from the cell [24]. Thus, the direct intra-oocyte [NO] measurements will help significantly decrease the distance from the point of generation, and produce more accurate and reproducible NO detection. To date, several concentrations of NO in different tissues have been reported. The [NO] measured in mouse ovary is approximately 1.4 times lower than the rate observed in oocytes obtained from young mice, but similar to the oocytes obtained from old animals [25]. The intra-oocyte [NO] is also lower than that reported for renal cortex and tubules [26], [27] which have been found to be ∼87 nM and ∼110 nM, respectively. Wakatsuki et al. and Malinski et al. have also reported the range of <0.5 nM to >1 µM for [NO] in brain tissue [28], [29]. Brovkoych et al. measured ∼140 nM in lung [24] and Hall et al. yielded concentration of 114–153 nM in cerebellar tissues [30]. NO plays a role as an inter-cellular and intra-cellular messenger in the range between 10 and 150 nmol/l [31], but after considering a wide range of [NO], we could conclude that NO functions physiologically in the nanomolar range (especially low nanomolar range) and micromolar volumes may be considered pathophysiological concentrations in cellular environments [32]. However, higher [NO] have been occasionally reported in cell membrane of endocardium (∼950 nM) [24] and smooth muscle cells (400–800 nM) [33].

Electrochemical methods are able to directly measure [NO] in tissues in situ. These methods function based on the electrochemical oxidation of NO on solid electrodes so current generated by NO oxidation is utilized as an analytic quantitative signal and can be measured in either amperometric or voltametric modes. The optimal capability of this method for direct measurement of NO is considered a huge milestone in NO research in biological components. In a recent study, Hunter et al. have challenged the accuracy of NO measurement techniques and reported significant variations between recorded measurements of [NO] in the same environments (i.e., solution or sample) [34]. It seems that there are potentially unknown factors in NO containing environments which affect the methods of NO detection in different manners. Similar applications could also be used successfully in oocytes from several species, perhaps with greater or lesser advantage, depending on oocyte dimensions, ease of microinjection etc. Nonetheless, in order to apply this technique to other types of cells, further refinement in the probe dimensions may be required. We feel that with advancement in this technology by miniaturization, this technique could be applied to a multitude of different cell types, expanding the horizons of NO-intracellular physiology.

In conclusion, for the first time this study introduces an oocyte-exclusive technique for measurement of [NO] which is aimed at minimizing interfering effects while preserving the highest sensitivity and accuracy in NO detection in a single cell complex, and will decrease complications of NO measurement, at least in the study of infertility.
